# Wnt/β‐catenin signaling: Structure, assembly and endocytosis of the signalosome

**DOI:** 10.1111/dgd.12718

**Published:** 2021-03-26

**Authors:** Gabriele Colozza, Bon‐Kyoung Koo

**Affiliations:** ^1^ Institute of Molecular Biotechnology of the Austrian Academy of Sciences (IMBA) Vienna Biocenter (VBC) Vienna Austria

**Keywords:** endocytosis, macropinocytosis, multivesicular endosomes, signalosome, Wnt/β‐catenin

## Abstract

Wnt/β‐catenin signaling is an ancient pathway that regulates key aspects of embryonic development, cell differentiation, proliferation, and adult stem cell homeostasis. Work from different laboratories has shed light on the molecular mechanisms underlying the Wnt pathway, including structural details of ligand–receptor interactions. One key aspect that has emerged from multiple studies is that endocytosis of the receptor complex plays a crucial role in fine‐tuning Wnt/β‐catenin signaling. Endocytosis is a key process involved in both activation as well as attenuation of Wnt signaling, but how this is regulated is still poorly understood. Importantly, recent findings show that Wnt also regulates central metabolic pathways such as the acquisition of nutrients through actin‐driven endocytic mechanisms. In this review, we propose that the Wnt pathway displays diverse characteristics that go beyond the regulation of gene expression, through a connection with the endocytic machinery.

## Graphical abstract

In this review, we analyze the role that endocytosis plays in regulating Wnt signaling. We also discuss recent findings on how Wnt regulates metabolic processes, through endocytic mechanisms.

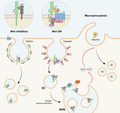

## 1 INTRODUCTION

Wnt signaling is an evolutionarily conserved pathway involved in embryonic development and cell differentiation (Nusse & Clevers, 2017). In addition, it regulates many other processes, including cell growth and mitosis, adult stem cell homeostasis, and regeneration (Acebron & Niehrs, 2016; Nusse & Clevers, 2017; Reddien, 2018). Aberrant activation of the Wnt pathway lies at the basis of different types of malignancies, including colorectal, breast, and hepatic cancers. Historically, the first Wnt gene was identified in *Drosophila melanogaster*, following a screen for recessive mutant genes that affect segment patterning of fruit fly embryos. Because the mutant flies did not develop wings, the gene was named *wingless* (*wg*) (Sharma, 1973; Sharma & Chopra, 1976). Years later, the proto‐oncogene *integration site‐1* (*int‐1*) was discovered while studying mouse models of mammary carcinomas induced by DNA integrations of the mouse mammary tumor virus (MMTV) (Nusse & Varmus, 1982). Since Int‐1 was found to be the mammalian orthologue of Wg, it was then renamed Wnt1 (Wingless + Int1) (Nusse et al., 1991).

Wnts are secreted glycoproteins (Brown et al., 1987; Papkoff et al., 1987) that can activate divergent pathways, classically categorized as: (a) the canonical or β‐catenin dependent pathway; and (b) the noncanonical branch, comprising the planar cell polarity and Ca^2+^ pathways. Although some Wnts preferentially activate one or the other pathway, their specificity is less strict than previously thought and likely depends on the cell‐specific context and the repertoire of receptors and components expressed, rather than on intrinsic properties. Indeed, while the serpentine Frizzled (Fzd) receptors are a shared component of all Wnt pathways, coreceptors such as the low‐density lipoprotein receptor‐related protein 5 and 6 (Lrp5/6; Arrow in *Drosophila*) (Joiner et al., 2013; Pinson et al., 2000; Tamai et al., 2000; Wehrli et al., 2000) or the Ror receptor tyrosine kinases (RTKs) (Green et al., 2014; Hikasa et al., 2002; Oishi et al., 2003) can confer specificity by driving canonical or noncanonical responses, respectively. With the identification of many components and regulators, much progress has been made towards an understanding of the molecular mechanisms underlying Wnt signaling. These basic aspects of the Wnt/β‐catenin pathway have been the subject of several excellent reviews (Cruciat & Niehrs, 2013; MacDonald et al., 2009; Niehrs, 2012; Nusse & Clevers, 2017).

On the other hand, in the last two decades, it also became evident that endocytosis plays a fundamental role in regulating Wnt signaling, yet the mechanistic link between Wnt and the endosomal machinery remains incompletely understood. Thus, an updated analysis revisiting our current understanding of the endosomal regulation of Wnt signaling is timely and appropriate. In this review, we will first discuss the main components of the Wnt/β‐catenin signaling machinery and how they assemble to form a fundamental signaling unit, the signalosome. We will provide biochemical and structural details that are fundamental for understanding the process of signalosome assembly. We will also discuss how signalosome formation is followed by endocytosis, and how this process triggers Wnt signaling through glycogen synthase kinase 3 (GSK3) inhibition. Finally, we will cover the most recent and unexpected findings showing that Wnt can modulate cell metabolism and nutrient acquisition through macropinocytosis.

## 2 THE MECHANISMS OF WNT/β‐CATENIN SIGNALING: AN OVERVIEW

**FIGURE 1:**
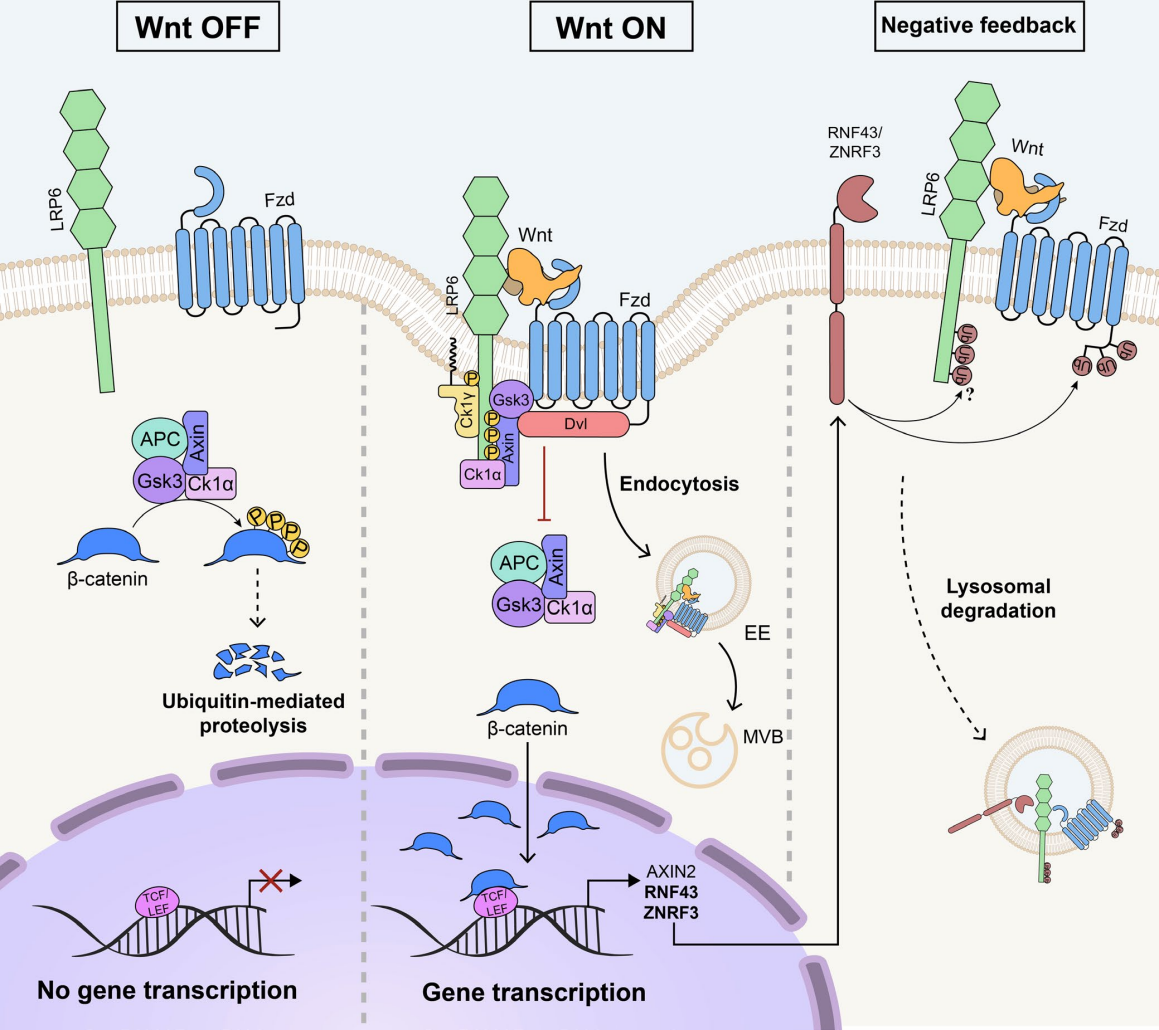
General model of Wnt/β‐catenin signaling. In absence of Wnt ligands, a destruction complex formed by Axin, APC, GSK3, and CK1 actively promotes β‐catenin protein turnover through a proteasome‐dependent mechanism, maintaining Wnt signaling in an OFF state. Conversely, in the Wnt ON state, a Wnt ligand binds to its cognate receptors, Fzd and Lrp5/6, inducing the formation of a multiprotein complex known as signalosome and inhibiting the destruction complex activity. The signalosome is subsequently endocytosed into early endosomes (EE), which later mature into multivesicular bodies (MVBs). Signalosome endocytosis is required to transduce the Wnt signal. Consequently, β‐catenin protein is stabilized and translocates into the nucleus, where together with TCF/LEF it activates the transcription of Wnt target genes. Among genes activated by β‐catenin/TCF, the transmembrane E3 ligases RNF43/ZNRF3 represent elements of an important negative feedback mechanism, which attenuate Wnt signaling by ubiquitination and degradation of the Fzd/Lrp5/6 receptor complex via the endolysosomal system. Note that, while Lrp6 stability is also regulated by RNF43/ZNRF3, it is still unclear whether it is directly ubiquitinated by the transmembrane E3 ligases

In the 30 years following its discovery (Nusse & Varmus, 1982, 2012), the Wnt/β‐catenin signal transduction has been the subject of many studies, which have identified and characterized many components of the underlying molecular machinery in great detail. The canonical pathway relies on the stability of β‐catenin (the vertebrate homolog of *Drosophila* Armadillo), a component of adherens junctions that is involved in the activation of Wnt transcriptional targets (Peifer, 1993, 1995; Peifer et al., 1991; Peifer & Wieschaus, 1990; van der Wal & van Amerongen, 2020). In the absence of ligands, the tumor suppressors Axin and adenomatous polyposis coli (APC) form a so‐called ‘destruction complex’ containing GSK3β (Kishida et al., 1998; Rubinfeld et al., 1996), which together with casein kinase 1 α (CK1α) phosphorylates β‐catenin sequentially (Figure 1) (Stamos & Weis, 2013). CK1α phosphorylates β‐catenin at Ser45, priming the consequent phosphorylation at Thr41, Ser37, and Ser33 by GSK3 (Liu et al., 2002; Wu & He, 2006). Axin greatly enhances β‐catenin phosphorylation by placing it in the vicinity of GSK3 (Ikeda et al., 1998). The β‐catenin phosphodegron is then recognized by β‐transducin repeats‐containing protein (β‐TrCP), the substrate‐recognition subunit of the E3 ubiquitin ligase Skp1–Cullin1–F‐box (SCF) protein complex, which promotes rapid turnover of β‐catenin through proteasome‐mediated degradation (Figure 1) (Fuchs et al., 2004; MacDonald et al., 2009).

Conversely, signaling is triggered when a Wnt ligand engages its cognate receptors, the seven‐pass transmembrane protein Fzd and the low‐density lipoprotein receptor‐related protein 5/6 (Lrp5/6). Binding to the receptors promotes the recruitment of several downstream effectors such as the adaptor Dishevelled (Dvl in mammals, Dsh in *Drosophila*), CK1γ, Axin1, and GSK3β (Davidson et al., 2005; Del Valle‐Perez et al., 2011), ultimately inhibiting the activity of the destruction complex. This allows the stabilization of β‐catenin protein, which then shuttles into the nucleus and activates the transcription of Wnt‐target genes upon binding to members of the DNA‐associated T‐cell factor/lymphoid enhancer‐binding factor (TCF/LEF) family of transcription factors (Figure 1) (Behrens et al., 1996; Huber et al., 1996; Molenaar et al., 1996; van de Wetering et al., 1991). Over the years, many additional players and regulatory mechanisms have been identified at all levels of Wnt signal activation. The following sections will review the different steps of the Wnt pathway in more detail.

## 3 STRUCTURAL INSIGHTS INTO WNT‐RECEPTOR BINDING

Wnts belong to a family of secreted glycoproteins conserved across metazoans, including cnidarians and platyhelminthes. In total, 19 different genes encoding for Wnt ligands have been identified in the human and mouse genome, while the hydra carries 13 (Willert & Nusse, 2012). Beside glycosylation, Wnt proteins feature a covalent attachment of palmitoleic acid, a monounsaturated fatty acid that renders the protein hydrophobic and poorly soluble in aqueous solutions (Willert et al., 2003). Palmitoylation occurs at the hydroxyl group of a conserved serine residue (e.g., S209 in Wnt3a and S187 in Wnt8) (Janda et al., 2012; Takada et al., 2006). The role of these posttranslational modifications in Wnt signaling has been covered in depth by several reviews (Coudreuse & Korswagen, 2007; Hosseini et al., 2019; Janda & Garcia, 2015). The hydrophobicity conferred by the palmitate necessitated complicated structural and biochemical characterization of Wnt proteins, until methods for large‐scale Wnt production and purification were developed. These protocols are elaborate and require the use of detergents, such as CHAPS, or other expedients to mask the hydrophobic group and maintain solubility (Willert et al., 2003; Willert, 2008).

**FIGURE 2:**
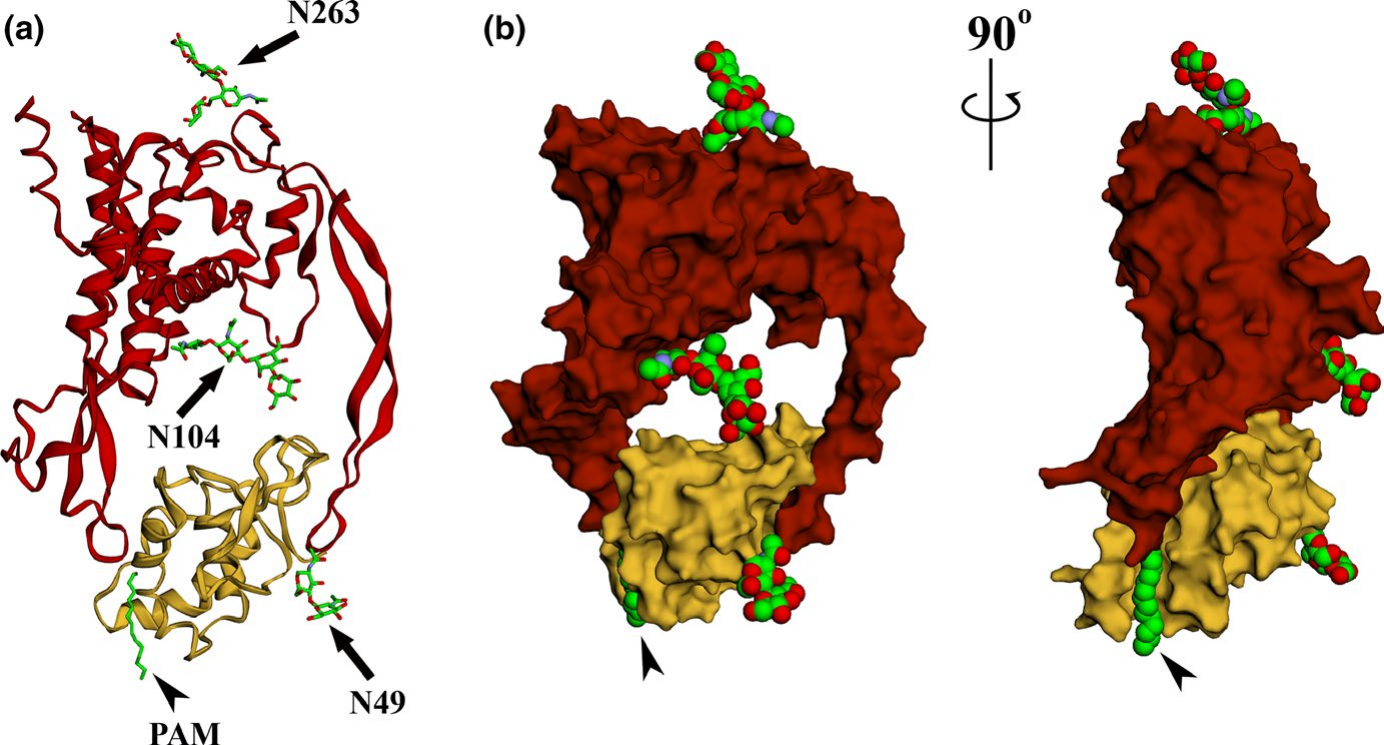
Crystal structure of the Wnt‐Frizzled complex. Wnt adopts a “thumb and index” structure that contacts Frizzled ectodomain on two opposite sides. (a) Ribbon models of xWnt8 (red) and Frizzled 8 cysteine rich domain (yellow). The palmitoleic acid moiety (PAM) and asparagine‐linked glycans are drawn as sticks, with the following atom color code: green = carbon; red = oxygen; blue = nitrogen. The black arrowhead points at the appended PAM extending in a zigzag pattern from Ser187 of the xWnt8 N‐terminal domain. Black arrows indicate Asn104‐linked glycan (two N‐acetylglucosamine and two mannose residues), Asn263‐linked glycan (two *N*‐acetylglucosamine residues and one mannose residue) on xWnt8; and Asn49‐linked glycan (two *N*‐acetylglucosamine residues) on Frizzled CRD. (b) Surface representation of the Wnt‐Fzd complex, ‘face‐on’ and ‘side‐on’ (after a 90 degrees rotation). Note how the palmitoleic acid (arrowheads) fits into the hydrophobic groove of Frizzled CRD. The images were created by uploading the crystallographic data of xWnt8‐Fzd8 complex deposited on Protein Data Bank (PDB ID 4F0A; https://www.rcsb.org/) (Janda et al., 2012), to the web‐based online tool EzMol (http://www.sbg.bio.ic.ac.uk/ezmol/) (Reynolds et al., 2018)

In 2012, a landmark crystallographic study determined for the first time the structure of a Wnt, *Xenopus* Wnt8 (xWnt8), in complex with the Fzd cysteine‐rich domain (CRD) at a resolution of 3.25 Å (Janda et al., 2012). A key finding was that coexpression of xWnt8 with Fzd8‐CRD allowed efficient purification of Wnt/Fzd complexes in the absence of detergents, suggesting that Fzd ectodomain could shield Wnt hydrophobicity. This study evidenced a novel type of structure not present in other secreted proteins, comparable to the palm of a hand, with thumb and index fingers that grasp the globular CRD in the extracellular region of Fzd (Figure 2a,b). Approximately two‐thirds of the xWnt8 sequence constitute the N‐terminal domain (NTD), which comprises the palm, featuring a seven α‐helical bundle, and the thumb domain containing the palmitoleic acid. Interestingly, the palmitoleate chain fits into a hydrophobic groove internal to the CRD, called site 1, and works as a sort of linchpin that strengthens the binding to Fzd receptors. Additional studies suggest that the Wnt lipid moiety traverses the entire groove, whose structure is flexible and adopts a U‐shape conformation that accommodates the kinked unsaturated fatty acid. The lipid eventually sticks its tip into the groove of an adjacent CRD, linking two Fzd receptors (Hirai et al., 2019; Nile et al., 2017). Fzd dimerization is further stabilized by the presence of a helical dimer interface, located near the lipid‐binding cavity of the CRD.

The remaining portion of Wnt, containing a high number of disulfide bridges, forms the C‐terminal index (or CTD, C‐terminal domain) that establishes hydrophobic bonds with key amino‐acid residues of Fzd site 2. Some of these site 2 contact residues are substituted in other Fzd receptors, likely providing some degree of discrimination for different Wnt ligands (Janda et al., 2012). All the 22 cysteines in the xWnt8 crystal structure were engaged in disulfide bridges, confirming that serine is the only residue modified by acylation. Notably, the linker region between the NTD and the CTD offers binding sites for specific coreceptors, as is the case for Wnt3a linker that binds to Lrp6 (Chu et al., 2013). In addition, a patch of 10 residues from three discontinuous regions was found conserved in several Wnts, and mapped to a solvent‐exposed surface opposed to the Fzd binding sites. The position and conservation of this patch suggest it might be involved in binding of coreceptors, like the linker region (Janda et al., 2012).

The structure of the coreceptor Lrp6 ectodomain has also been resolved by combining crystallography and negative stain electron microscopy, revealing a compact horseshoe‐like structure (Chen et al., 2011). The ectodomain of Lrp5/6 is organized in tandem repeats of four Tyr‐Trp‐Thr‐Asp (YWTD) β‐propeller–EGF domains (E1–E4), followed by three LDLR type A (L1‐L3) domains and the transmembrane segment. Binding to Wnt ligands, agonists and antagonists occurs mostly in the E1–E4 region (He et al., 2004). There is evidence that E1–E2 and E3–E4 pairs are relatively rigid blocks, connected by a short hinge (Cheng et al., 2011), and serve as functional units for the binding of distinct ligands. For example, Wnt1, xWnt8, and Wnt9b bind the first two propellers and Wnt3a binds propellers 3 and 4, while the Wnt antagonist Dkk1 binds both E1–E2 and E3–E4 (Bourhis et al., 2010; Ettenberg et al., 2010; Glinka et al., 1998; Itasaki et al., 2003; Mao et al., 2001). Binding occurs through key residues on the top surfaces of the propeller domains of Lrp5/6, whose mutations are the cause of several syndromes, including autosomal dominant high bone mass (HBM) (Ai et al., 2005; Boyden et al., 2002; Chen et al., 2011; Cheng et al., 2011). The presence of overlapping binding surfaces for Wnts and Dkk1 provides support for inhibition by direct competition, in parallel to other antagonistic mechanisms (Semenov et al., 2001, 2008).

## 4 INITIATING WNT SIGNALING: ASSEMBLY OF THE SIGNALOSOME

Upon binding to Wnt ligands, cognate receptors Fzd and Lrp6 are bridged together and coalesce into multiprotein complexes, termed Wnt signalosomes, which appear as discrete puncta at or below the plasma membrane (Bilic et al., 2007). A recent study suggests that sulfate‐rich heparan sulfate proteoglycans (HSPG) are involved in the formation of the signalosome. Specifically, *N*‐sulfo‐rich glypicans form clusters that colocalize with Wnt8/Fzd/Lrp6 puncta in vivo in *Xenopus* embryos, acting as recruiting platforms that accumulate Wnt ligands and promote the assembly of Wnt signalosomes (Mii et al., 2017). Notably, glypicans of the Dlp (Dally‐like protein) family have been recently shown to bind the Wnt palmitoleate through a hydrophobic tunnel in their core protein, shielding the lipid moiety from the aqueous environment and serving as reservoirs that hand Wnt proteins to signaling receptors (McGough et al., 2020). Furthermore, the presence of certain coreceptors may promote the enrichment of specific Wnt ligands within signalosomes, in a context‐specific fashion. For example, the glycosylphosphatidylinositol (GPI)‐anchored protein Reck binds specifically to the intrinsically disordered linker region of Wnt7a and b, and together with the G‐protein coupled receptor Gpr124 promotes the formation of Lrp6/Fzd signalosomes in response to Wnt7 ligands (Cho et al., 2019; Eubelen et al., 2018; Vallon et al., 2018). This ligand‐selective mechanism of signalosome activation is crucial for central nervous system angiogenesis.

After initiation, multiple signalosomes may associate into higher‐order Lrp6‐Fzd oligomers with potentiated signaling activity. Clustering between Fzd‐Lrp6 units is favored by the presence of Tmem59, a single‐pass transmembrane protein that interacts with Fzd and has a high tendency to self‐associate (Gerlach et al., 2018). In addition, multisite interactions between Fzd and Lrp5/6 can occur ligand independently and promote heterooligomerization of receptor complexes (Hua et al., 2018). It is still incompletely understood how the signalosome unit is assembled, but key downstream components, such as Dvl, Axin, GSK3, and CK1s are known to regulate the formation of these complexes. For example, the coordinated activity of multiple CK1 isoforms plays an essential role, through a cascade of phosphorylations that promote the binding or activity of several Wnt components within the receptor complex (Del Valle‐Perez et al., 2011). One of the earliest responses following ligand stimulation is the phosphorylation of the Lrp6 intracellular domain (Bilic et al., 2007; Davidson et al., 2005; Tamai et al., 2000, 2004; Zeng et al., 2005). Lrp6 phosphorylation occurs at serine/threonine residues contained in the PPP(S/T)P motifs, which are reiterated five times in the Lrp5/6 carboxy terminus, and is mediated by the proline‐directed kinase GSK3 (Zeng et al., 2005). Replacement of these serine/threonine residues with alanine (which cannot be phosphorylated) impairs signal transduction. PPP(S/T)P phosphorylation serves as the priming site for the sequential phosphorylation of adjacent S/T residues, by CK1α and δ (Zeng et al., 2005). These studies revealed a surprising function for GSK3 in activating Wnt signaling at the receptor level, distinct from its classic inhibitory role in the cytosolic destruction complex (Zeng et al., 2005). In addition, CK1γ has also been shown to phosphorylate Lrp6 in S/T clusters near the primed PPP(S/T)P sites following Wnt stimulation (Davidson et al., 2005). CK1γ is a unique member of the casein kinase family in that it is anchored to the plasma membrane through a C‐terminal palmitoylation, and is required for Wnt signaling, as CK1γ loss of function abolishes Wnt activation both in cell cultures and *Xenopus* embryos (Davidson et al., 2005).

Formation of the signalosome, as well as Lrp6 phosphorylation (both GSK3‐ and CK1γ‐ mediated), depends on the cytosolic adaptor Dvl, which is relocated to the plasma membrane and forms a complex with Fzd early after Wnt stimulation (Bilic et al., 2007; Casagolda et al., 2010; Wong et al., 2003; Zeng et al., 2008). Binding and relocalization of Dvl requires conserved residues located on the Fzd intracellular loops, as well as a short amino acid sequence (Lys‐Thr‐X‐X‐X‐Trp) on the Fzd C‐terminal tail (Cong et al., 2004; Umbhauer et al., 2000). This interaction may be stabilized via CK1ε, which is responsible for Dvl phosphorylation (Bernatik et al., 2011; Casagolda et al., 2010; Del Valle‐Perez et al., 2011; Gonzalez‐Sancho et al., 2013; Peters et al., 1999). Recent single‐molecule analysis revealed the precise dynamics of endogenous Dvl at the plasma membrane. In the absence of Wnt ligands, monomeric Dvl shuttles on and off the plasma membrane with a dwell time of <1 s. After Wnt stimulation, however, increased affinity for Fzd raises local Dvl concentration, driving the formation of Dvl oligomers with higher retention time (2–3 min) at the plasma membrane (Ma et al., 2020). Dvl DIX domain is instrumental for forming these dynamic and reversible head‐to‐tail oligomers, which ultimately increase the binding sites for signaling effectors and promote the assembly of Wnt signalosomes (Schwarz‐Romond, Fiedler, et al., 2007). The Dvl DEP domain is also important as it dimerizes upon binding to Fzd, likely crosslinking multiple signalosomes (Gammons et al., 2016; Gammons et al., 2016).

The net result of Lrp6 phosphorylation is the recruitment of the tumor suppressor Axin. Axin is initially brought to the receptor by Dvl, through dynamic interactions between their DIX domains (Cliffe et al., 2003; Schwarz‐Romond, Fiedler, et al., 2007; Schwarz‐Romond et al., 2007). Since Axin is complexed with GSK3 and CK1, its recruitment to the signalosome may increase the local concentration of these enzymes in proximity of Lrp6, thus favoring phosphorylation of the coreceptor C‐terminal tail, while it dissociates the destruction complex for β‐catenin degradation. Indeed, reduction in Axin levels or an Axin mutant incapable of binding GSK3 (Axin L396Q, or *masterblind* in zebrafish) prevents Lrp6 phosphorylation (Heisenberg et al., 2001; van de Water et al., 2001; Zeng et al., 2008). In turn, the phosphorylated C‐terminal domain of Lrp6 provides a docking platform that reinforces Axin and GSK3 association with the receptor complex. In agreement with this, phosphomutant Lrp6 shows much reduced binding to Axin (Davidson et al., 2005; Zeng et al., 2005). The interaction with phospho‐Lrp6 inhibits GSK3 activity, as indicated by the decrease of GSK3‐mediated phosphorylation of β‐catenin, which consequently leads to β‐catenin protein stabilization (Cselenyi et al., 2008; Mi et al., 2006; Piao et al., 2008; Wu et al., 2009; Zeng et al., ,^2005^, ^2008^).

While Lrp6 Ser/Thr phosphorylation induced by Wnt is required for signaling activation, Lrp6 Tyr phosphorylation serves as a mechanism to turn off the Wnt pathway. A cDNA screening identified the nonreceptor tyrosine kinases Src and Fer as Lrp6 modifiers (Chen et al., 2014). In contrast to Ser/Thr kinases, Src and Fer were shown to inhibit Wnt, by reducing Lrp6 levels and disrupting signalosome formation. Interestingly, Src/Fer‐mediated phosphorylation of conserved tyrosine residues next to the PPP(S/T)P clusters was promoted by Wnt stimulation, thus initiating a rapid negative feedback that prevents overactivation of Wnt signaling (Chen et al., 2014).

## 5 ENDOCYTOSIS OF THE WNT SIGNALOSOME

What happens to the activated mature signalosome? Endocytosis has long been known as an essential requirement for proper Wnt signal transduction, and there are now multiple lines of evidence showing that the signalosome is internalized by endocytosis following Wnt stimulation. The earliest evidence that endocytosis promotes Wnt signaling came from in vivo studies on *Drosophila melanogaster*. Accordingly, Wg was found in a punctate pattern on receiving cells in the developing wing, and Wg puncta disappeared when endocytosis was blocked (Strigini & Cohen, 2000; van den Heuvel et al., 1989). Importantly, the *Drosophila* Lrp5/6 orthologue Arrow and Fzd were internalized as well, together with Wg (Rives et al., 2006). Trafficking of the Wg/receptor complex to early endosomes was required for proper wing disc patterning, as blocking endocytosis through knockdown of *shibire* (the *Drosophila* orthologue of dynamin, a GTPase that pinches the forming endocytic vesicles off the plasma membrane) or Rab5 (a GTPase involved in endosomal maturation), markedly reduced Wg target transcription in vivo (Seto & Bellen, 2006). Vesicular puncta containing Wg and Dsh colocalized with several endosome‐associated proteins, such as Rab7, Arrestin, and benchwarmer/spinster (Seto & Bellen, 2006), an endolysosomal sugar carrier (Dermaut et al., 2005).

Like in *Drosophila*, Wnt signaling in mammalian cells also depends on endocytic activity. In fact, Lrp6 internalization in cultured cells occurs as early as 10 min after stimulation with Wnt3a, peaking after around 1–2 hr of treatment (Yamamoto et al., 2006). Together with Lrp6, Fzd and Axin are also recruited to intracellular vesicles in a Wnt‐dependent manner (Yamamoto et al., 2006). Despite the evidence gathered from *Drosophila*, it is still debated whether Dvl also traffics on endosomal vesicles following Wnt activation. In earlier works, Dvl was found on vesicle‐like organelles in mammalian cultured cells, as well as frog embryos and animal cap explants, and this localization was dependent on the presence of a phospholipid‐binding motif (VKEEIS) in its DIX domain (Axelrod et al., 1998; Capelluto et al., 2002; Miller et al., 1999). Costaining with concanavalin A (a lectin that binds glycoproteins on membrane‐bound vesicles) and the affinity for the micelle‐forming phospholipid mimic dodecylphosphocholine (DPC) (Capelluto et al., 2002) led to the conclusion that Dvl interacts with some type of cytoplasmic vesicles, the identity of which remained elusive. However, other studies found no evidence of colocalization between Dvl and known endosomal markers and suggested that the vesicle‐like puncta commonly observed upon Dvl overexpression actually represent protein aggregates that form dynamically in the absence of vesicle membranes, as a consequence of Dvl’s ability to coalesce and multimerize (Schwarz‐Romond et al., 2005; Smalley et al., 2005). Despite the contrasting reports, overexpressed Dvl puncta were later found to overlap with GFP‐FYVE (Taelman et al., 2010), a reagent that recognizes a key endosomal component, phosphatidylinositol 3‐phosphate (PI3P), and endogenous Dvl‐2 was also found associated with cytoplasmic membrane‐bound organelles after stimulation with Wnt3a or constitutively active Lrp6 (Vinyoles et al., 2014).

Thus, following Wnt signaling activation, the Wnt signalosome and its associated components translocate into endosomal vesicles. This property might also be a conserved feature of β‐catenin independent branches of Wnt signaling, since it has recently been observed that several Wg components, including Fzd, Axin, and Dsh, localized on Rab5^+^ endosomes and were required for microtubule nucleation at dendrite branch points in *Drosophila* neurons, following an “apocryphal” pathway that does not rely on Armadillo stabilization (Weiner et al., 2020).

## 6 ENDOCYTIC ROUTES FOR WNT SIGNALING

**FIGURE 3:**
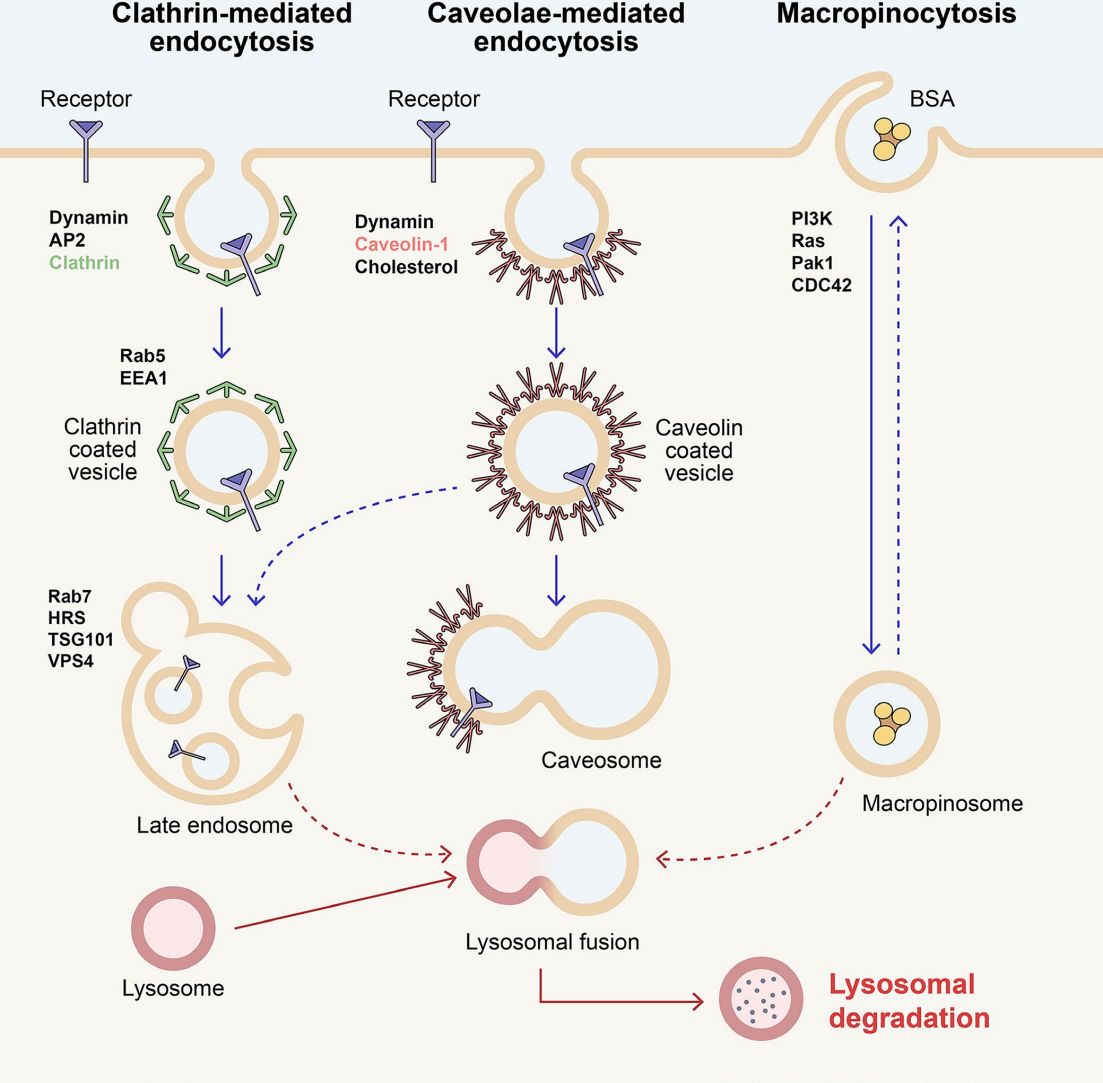
Cellular mechanisms of endocytosis. The diagram shows clathrin‐mediated endocytosis, caveolar endocytosis, and macropinocytosis, all of which have pertinence to Wnt signaling. The major regulatory proteins for the different endocytic processes are indicated. Although these pathways proceed through different mechanisms, they converge at the level of the late endosomal/lysosomal system. Note that macropinocytosis is a receptor‐independent pathway, different from clathrin‐ and caveolin‐dependent endocytosis

Endocytosis occurs through distinct, well‐characterized pathways (Doherty & McMahon, 2009), including clathrin‐mediated endocytosis (CME) (Mettlen et al., 2018), caveolar endocytosis (Parton et al., 2020), and macropinocytosis (King & Kay, 2019) (Figure 3). Regarding the specific endocytic pathway required for Wnt signaling, there have been conflicting reports so far. Caveolin, a protein found in caveolae (Rothberg et al., 1992), was initially found to overlap with vesicles containing the Lrp6 signalosome (Bilic et al., 2007; Yamamoto et al., 2006, 2008). Caveolae are flask‐shaped invaginations of the plasma membrane, containing detergent‐resistant microdomains (DRMs) rich in cholesterol and glycosphingolipids, and are coated with caveolin‐1 oligomers (Hooper, 1999; Parton et al., 2020). Lrp6 palmitoylation at Cys^1394^ and Cys^1399^ promotes enrichment of the coreceptor in DRMs (Sada et al., 2019). Knockdown of caveolin, but not clathrin, inhibited Lrp6‐signalosome endocytosis and Wnt‐induced β‐catenin stabilization in HEK293 and HeLa cells, and inhibition of caveolae formation with cholesterol‐binding drugs (Table 1) had similar effects (Yamamoto et al., 2006), indicating that canonical Wnt depends on caveolar endocytosis (Yamamoto et al., 2008). Lrp6 caveolar endocytosis also requires the small GTPase Rab8B, a regulator of intracellular vesicle trafficking that interacts with Lrp6 and its activator CK1γ, and colocalizes with Dvl‐1 in cytoplasmic puncta. Knockdown of Rab8B, or overexpression of a dominant‐negative, abrogated Lrp6 internalization and reduced Wnt signaling (Demir et al., 2013).

**TABLE 1:** List of endocytic inhibitors known to affect Wnt/β‐catenin signaling

Drug	Target	Effect on Wnt	System used	References
Apicularen	Vacuolar ATPase inhibitor	Inhibition	HEK293T cells	Cruciat et al. (2010)
Bafilomycin	Vacuolar ATPase inhibitor	Activation (low dosage)/Inhibition (high dosage)	HEK293T cells	Dobrowolski et al., (2012); Cruciat et al. (2010)
Chloroquine	Lysosomal and autophagy inhibitor	Activation (low dosage)/Inhibition (high dosage)	HEK293T cells	Dobrowolski et al. (2012)
Chlorpromazine (CPZ)	Clathrin endocytosis inhibitor	Inhibition	murine Lcells	Blitzer and Nusse (2006)
Dynasore	Dynamin inhibitor	Inhibition	*Drosophila* S2R+ cells	Gagliardi et al. (2014)
Dyngo−4a	Dynamin inhibitor	Inhibition	*Drosophila* S2R+ cells, RKO cells	Gagliardi et al. (2014)
E64	Lysosomal protease inhibitor	Activation	LSL cells	Dobrowolski et al. (2012)
EIPA/Amiloride	Macropinocytosis inhibitors	Inhibition	SW480, HEK293T	Tejeda‐Muñoz et al. (2019)
Filipin III	Caveolar endocytosis inhibitor	Inhibition	HeLa, CHO cells	Yamamoto et al. (2006)
Leupeptin	Lysosomal protease inhibitor	Activation	LSL cells	Dobrowolski et al. (2012)
Monodansylcadaverin (MDC)	Clathrin endocytosis inhibitor	Inhibition	murine L cells	Blitzer and Nusse (2006)
Nystatin	Caveolar endocytosis inhibitor	Inhibition	HeLa, CHO, RKO cells	Yamamoto et al. (2006); Saito‐Diaz et al. (2018)
Pitstop−2	Clathrin endocytosis inhibitor	Inhibition	RKO (APC‐) cells; APCmin mouse intestinal organoids	Saito‐Diaz et al. (2018)

However, at the same time it was reported that multiple clathrin inhibitors (Table 1), as well as siRNA‐mediated depletion of clathrin, impaired Wnt signaling in murine L cells (Blitzer & Nusse, 2006). Clathrin and its associated adaptor protein 2 (AP2) were also required in other cell lines, such as HEK293 and mouse embryonic fibroblasts (MEF). Knockdown of clathrin or AP2 blocked Wnt signaling in these cells, by impairing the formation of Lrp6 signalosomes at the plasma membrane and reducing Sp1490 Lrp6 phosphorylation (Kim et al., 2013). In this study, however, Wnt3a‐dependent Lrp6 internalization was observed only upon hydrolysis of phosphatidylinositol (4,5)bisphosphate (PtdIns(4,5)P2). A similar dependence on clathrin and AP2 for signalosome formation and signaling was also found in zebrafish (Hagemann et al., 2014).

What are the factors that determine the specific endocytic route for signalosome internalization? First, the Lrp6 receptor tends to spontaneously undergo clathrin‐mediated endocytosis in the absence of ligand stimulation. Elegant work from Ethan Lee’s lab has recently demonstrated that APC inhibits Lrp6 signalosome internalization in the absence of Wnt ligand. This activity, which is specific to APC but not APC2, prevents inadvertent activation of the Wnt pathway (Saito‐Diaz et al., 2018). When APC is inactivated, Lrp6 is rapidly internalized through the clathrin pathway, as demonstrated by immunofluorescence on colorectal cancer cells with APC truncation (Saito‐Diaz et al., 2018). However, when a ligand is present, the predominant pathway regulating signalosome endocytosis may be dictated by the cell type. Indeed, it has been suggested that epithelial cells such as HEK293, RKO colorectal cancer, and retinal pigmented epithelium (RPE) cells utilize mainly the caveolin pathway, while fibroblasts (MEFs, L cells) use clathrin endocytosis (Saito‐Diaz et al., 2018). As many of these studies relied on the overexpression of single Wnt pathway components, stoichiometry may also play a role; in fact, when overexpressed alone, Fzd was endocytosed through clathrin‐coated vesicles upon Wnt stimulation. However, when Lrp6 and Fzd were coexpressed, caveolin endocytosis prevailed (Yamamoto et al., 2006).

Another possibility is that signals present on the receptors can also influence what pathway mediates the internalization process. For example, in the case of CME, interaction between AP2 and its endocytic cargo requires the latter to possess an internalization motif, YXXØ (where Y is a Tyr, X any amino acid, and Ø is a bulky hydrophobic residue such as Leu, Ile, Phe, Met, or Val) (Ohno et al., 1998; Traub & Bonifacino, 2013). Of note, Lrp6 has two consecutive Tyr‐based motifs in the cytoplasmic C‐terminal region, starting at Tyr‐1517 and Tyr‐1522 (YRPY and YRHF sequences, conserved from *Homo sapiens* to *Xenopus laevis*). Accordingly, Y1522A mutation blocked Lrp6–AP2 interaction and decreased Wnt‐induced reporter activity (Kim et al., 2013), and the same mutation prevented clathrin‐mediated Lrp6 internalization in HepG2 hepatic cells (Yamamoto et al., 2017), a process required for Lrp6‐dependent clearance of low‐density lipoproteins (LDL). Conversely, mutations of both Tyr‐based endocytic sequences were shown to increase Lrp6 distribution in caveolar domains and augment Wnt activation in HEK293 cells, likely reflecting the preference for caveolin endocytic route in this cell line (Liu et al., 2014). Because the AP2 tyrosine‐interacting pocket cannot accommodate phosphotyrosines (Shiratori et al., 1997), tyrosine phosphorylation in the YXXØ motifs serves as a simple, yet effective, mechanism to modulate endocytosis by preventing binding to AP2. Currently, it is unclear if Lrp6 internalization is modulated by Y‐1517 or Y‐1522 phosphorylation or similar mechanisms.

## 7 ADDITIONAL FACTORS REQUIRED FOR SIGNALOSOME ENDOCYTOSIS

During the last few years, several labs have revealed additional links between factors involved in endosome trafficking and maturation, and Wnt signaling. For instance, recent work has shown a role for Ral GTPases in signalosome internalization. RalA and RalB are Ras effectors involved in several processes, including endocytosis (Jiang et al., 2016; Jullien‐Flores et al., 2000). Both Ral genes were required for Wnt‐induced Fzd endocytosis in HEK293 cells, and their activity was critical for Wnt signaling (Johansson et al., 2019). RalA and B expression is prominent in *Drosophila* intestinal stem cells (ISCs) and in mouse intestinal crypts. Notably, Wnt signaling is high in the crypts and is fundamental for the proliferation and maintenance of ISCs (Clevers, 2013; Gehart & Clevers, 2019). Loss of RalA and B caused suppression of Wnt signaling in vivo in the mouse intestine, causing ISC depletion and intestinal crypt death (Johansson et al., 2019). Recent work found an interesting connection between Wnt/β‐catenin, epidermal growth factor receptor (EGFR), and endocytosis. In this study, Wnt9a and its cognate receptor Fzd9b were found to be key elements for hematopoietic stem cell development (Grainger et al., 2019). Elegant proximity labeling experiments using an ascorbate peroxidase (APEX)‐based biotinylation system (Hung et al., 2014; Lam et al., 2015) showed, unexpectedly, that EGFR was part of the Wnt9a‐Fzd9b complex (Grainger et al., 2019). Under Wnt stimulation, EGFR phosphorylated Fzd9b at Y556, promoting Wnt/β‐catenin signaling. Interestingly, the proximity labeling analysis also showed that the endosomal machinery and components of the clathrin pathway were recruited to the signalosome a few minutes after Wnt stimulation (Grainger et al., 2019). The clathrin endocytic pathway was pivotal for signal transduction, since its inhibition decreased Wnt signaling. Thus, the study from Grainger and colleagues supports a model where, in the presence of a Wnt ligand, EGFR joins the signalosome where it phosphorylates Fzd, promoting internalization of the receptor complex, followed by β‐catenin nuclear accumulation. It is currently unclear whether EGFR activity is specific for the Wnt9a/Fzd9b pair, and if it also promotes Wnt signalosome internalization and signaling in other cell types or developmental contexts.

Recently, we employed a proximity labeling approach like the one described by Grainger and colleagues, to study the protein network dynamics of Wnt signaling at the receptor level. Using an Lrp6‐APEX2 chimeric receptor expressed in HEK293T cells, we were able to identify the Lrp6 interactome (that is, the proteins recruited in proximity of Lrp6) in the presence or absence of Wnt3a ligand (Colozza et al., 2020). Our results showed that components of the endosomal sorting complex required for transport (ESCRT), particularly Hrs and Tsg‐101, were recruited as early as 5 min after ligand addition, indicating that the recruitment of the endosomes is a very early response to Wnt signaling, in agreement with Grainger et al. (2019). Interestingly, Trk‐fused gene (TFG) was found among the most enriched biotinylated proteins. TFG is a protein regulating secretion in the ER‐Golgi intermediate compartment (ERGIC) (Hanna et al., 2017; Johnson et al., 2015), and oncogenic fusions of TFG with Trk receptors are involved in thyroid cancer (Greco et al., ,^1992^, ^1998^; Roccato et al., 2003). A recent study has shown that TFG is also involved in regulating autophagic flux in CH12 B lymphoma cells (Steinmetz et al., 2020). The TFG N‐terminal end features a Phox‐Bem 1 (PB1) domain, which confers the ability to oligomerize. Interestingly, PB1 adopts a ubiquitin‐like β‐grasp fold, an evolutionarily conserved structure closely related to the DIX domain (Ehebauer & Arias, 2009; Yamanishi et al., 2019). Loss‐of‐function experiments in cultured cells and in vivo in *Xenopus* embryos showed that TFG is required for Wnt signaling (Colozza et al., 2020). Importantly, our study also revealed that TFG can localize on Hrs^+^ endosomal vesicles, but the mechanisms through which TFG regulates Wnt signaling are still unknown.

Endosomal acidification is also important for Wnt signaling. For example, prorenin receptor (PRR) was shown to be part of the signalosome, by interacting with Lrp6 and Fzd (Cruciat et al., 2010). PRR associated with the vacuolar ATPase (V‐ATPase) complex, responsible for the acidification and trafficking of endosomal vesicles (Collins & Forgac, 2020), thus working as a specific adaptor between the Wnt receptor and V‐ATPase complexes. Knockdown of either PRR or V‐ATPase subunits drastically reduced Wnt signaling (Cruciat et al., 2010). Interestingly, it was recently found that PRR expression is increased in colorectal cancer tissues and promotes cancer progression by potentiating Wnt signaling, in cooperation with other Wnt‐activating mutations (Wang et al., 2019). Similarly, TMEM9, a protein that associates with PRR and the V‐ATPase complex and stimulates endolysosomal acidification, was also found to upregulate Wnt signaling (Jung et al., 2018). TMEM9 levels were elevated in colorectal cancer and hepatocellular carcinoma cell lines, and contributed to tumorigenesis through Wnt signaling, in a V‐ATPase‐dependent manner (Jung et al., 2018, 2020). Interestingly, GSK3 inhibition with chemical inhibitors or siRNA was also found to enhance endolysosomal acidification through increased autophagic activity and endosomal maturation, in cultured cancer cells and mouse models for Alzheimer disease (Avrahami et al., 2013, 2020; Azoulay‐Alfaguter et al., 2015). Thus, it is possible that modulation of GSK3 activity by Wnt plays a key role in endosome acidification and biogenesis.

## 8 CONNECTING ENDOCYTOSIS TO WNT SIGNALING: THE SEQUESTRATION MODEL

**FIGURE 4:**
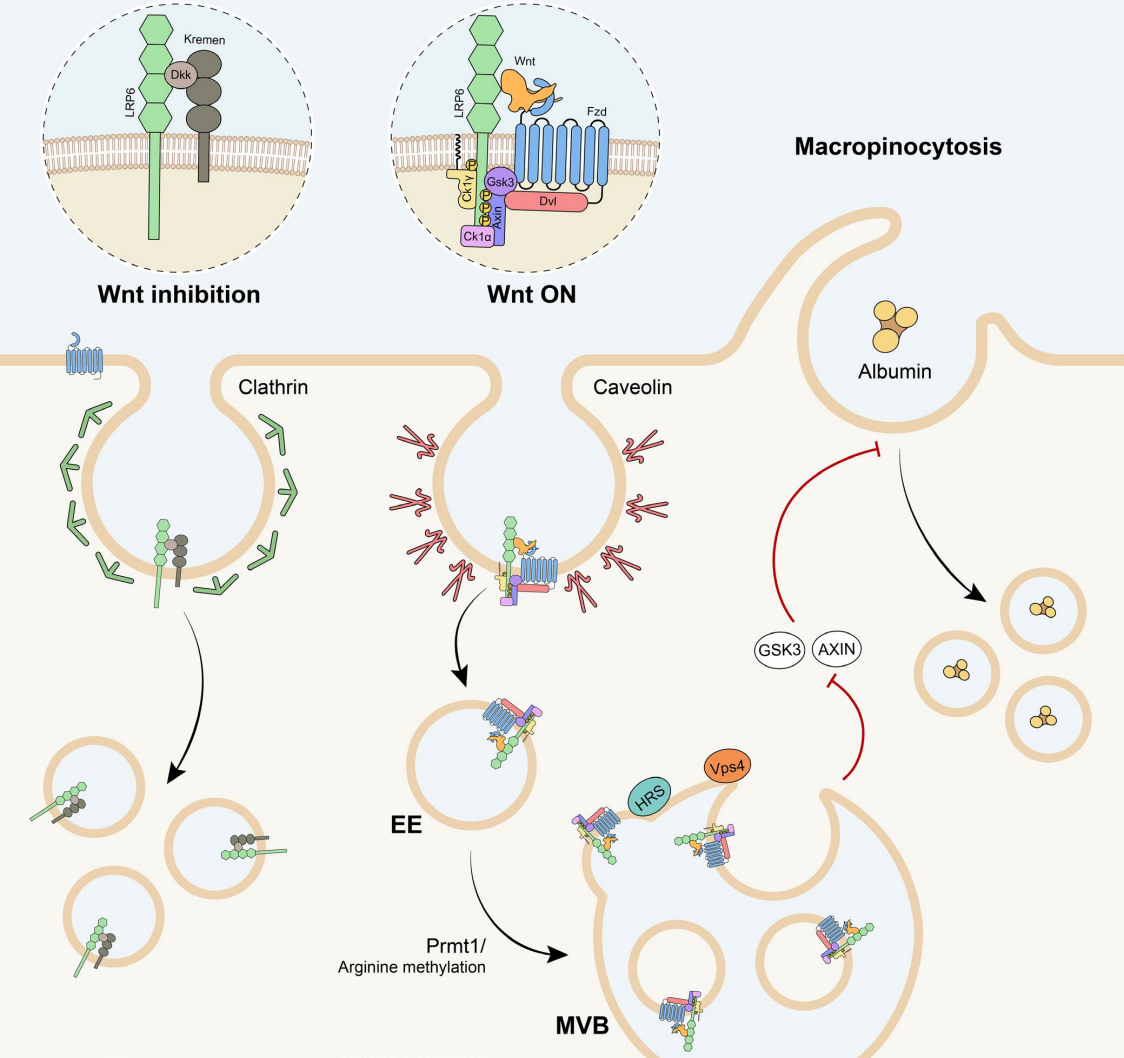
Endocytosis regulates Wnt signaling. Upon activation of the Wnt pathway, the signalosome (formed by the ligand–receptor complex, and associated proteins) is rapidly internalized through caveolar endocytosis. Early endosome (EE) vesicles containing the signalosome fuse and mature into multivesicular bodies (MVB), dragging GSK3 and Axin into the intraluminal vesicles of the MVB. Several endosomal proteins, such as the ESCRT machinery (including HRS and VPS4), are required for this process. Prmt1 and arginine methylation are also key regulators of GSK3 sequestration into MVBs. On the other hand, endocytosis can also be utilized to dampen Wnt signal. An example is Dkk1 binding to Lrp6 and the transmembrane protein Kremen, which promote Lrp6 clearance from the plasma membrane through clathrin‐mediated endocytosis. Wnt can also mediate nutrient uptake, by regulating macropinocytosis. GSK3 and Axin normally repress micropinocytosis; however, when Wnt signaling is turned on, their sequestration into MVBs allows for sustained macropinocytic activity

Further evidence that Wnt requires the endosomal compartment for proper signaling was reported in 2010. Using a combination of immunostaining, electron microscopy, and protease K protection assays on mammalian cells, the group of Eddy De Robertis showed that Wnt3a, or a constitutively active form of Lrp6, induced GSK3 internalization into endosomal organelles named multivesicular bodies (MVBs) (Dobrowolski & De Robertis, 2011; Taelman et al., 2010). Mechanistically, sequestration into membrane‐bound vesicles keeps GSK3 away from its cytosolic substrates, most notably β‐catenin, which then escapes degradation and activates Wnt target gene transcription in the nucleus (Figure 4). Consistent with this, knockdown of ESCRT machinery components, such as Hrs, or use of dominant‐negative mutant ESCRT proteins, such as Vps4 EQ, prevented endosomal relocalization of GSK3 and resulted in strong Wnt inhibition both in cell cultures and *Xenopus* embryos (Taelman et al., 2010). Presenilin deficiency or low doses of the lysosomal inhibitor chloroquine induced accumulation of multivesicular endosomes, increasing Wnt sensitivity in cultured cells and *Xenopus* embryos (Dobrowolski et al., 2012). The expanded endosomal compartment led to a more efficient GSK3 sequestration and, according to this, the increase in Wnt response in presenilin‐deficient cells required ESCRT‐dependent endosomal formation (Dobrowolski et al., 2012). Similar to GSK3, Lrp5/6, Dvl‐2, Axin, and even phospho‐β‐catenin were also found to move into endosomal vesicles, indicating that the whole signalosome protein complex is internalized by endocytosis (Vinyoles et al., 2014), in agreement with previous observations (Yamamoto et al., 2006). Interestingly, the signalosome endocytosis is regulated by p120‐catenin/cadherin, which tether Lrp6 to the plasma membrane. However, in the presence of Wnt ligands, p120‐catenin and cadherin are phosphorylated and released from the Lrp6/signalosome complex, which is then endocytosed (Vinyoles et al., 2014).

Despite much effort, how Wnt promotes ESCRT‐dependent GSK3 sequestration is still incompletely understood. Recent findings suggest that different mechanisms, including posttranslational modifications and autophagy, may play a fundamental role. For example, protein arginine methylation was found to be indispensable for Wnt‐induced GSK3 sequestration. Arginine methylation is mediated by a family of protein arginine methyltransferases (PRMTs), of which PRMT‐1 alone accounts for 85% of total protein arginine methylation (Bedford & Clarke, 2009; Tang et al., 2000). PRMT‐1 was previously shown to inhibit Wnt by methylating Dvl‐3 and interfering with its function (Bikkavilli et al., 2012), or through methylation of the scaffold protein Axin, a modification that stabilizes this negative regulator (Cha et al., 2011). However, recent works established a positive role for PRMT‐1 in Wnt regulation. PRMT‐1 loss of function, or pharmacological inhibition of methylation by adenosine‐2′,3′‐dialdehyde (Adox), impaired GSK3 relocalization into endosomes and reduced Wnt signaling in mammalian cells (Albrecht et al., 2018). Noteworthily, a physical interaction between PRMT‐1 and Lrp6 was previously detected using mass spectrometry analysis to find factors that regulate osteochondrogenic differentiation (Cheng et al., 2015). In addition, methylation of the Dvl‐binding protein G3BP2 (Ras GTPase activating protein‐binding protein 2) by different PRMTs, including PRMT‐1, was found to promote phosphorylation and activation of Lrp6 receptor (Bikkavilli & Malbon, 2012). Altogether, these data highlight an important role for arginine methylation during Wnt signaling.

It has been suggested that PRMT‐1 promotes GSK3 sequestration through ESCRT‐driven microautophagy (Albrecht et al., ,^2018^, ^2019^). Interestingly, *C. elegans* epg‐11, which encodes an arginine methyltransferase homologous to PRMT‐1, was observed to regulate P‐granule autophagy during embryonic development (Li et al., 2013). Thus, it is possible that PRMT‐mediated arginine methylation confers specificity for selective autophagy, and that this process is in part required for Wnt signaling. Supporting further the link between Wnt and autophagy, 3T3‐L1 preadipocytes were shown to incorporate GSK3 into MVBs in a process regulated by tumor protein p53‐inducible nuclear protein 2 (TP53INP2), a regulator of autophagy (Romero et al., 2018). Interestingly, GSK3 colocalized with typical autophagic markers, such as microtubule‐associated protein 1 light chain 3 (LC3‐II) inside multivesicular endosomes. GSK3 sequestration and Wnt pathway activation required the integrity and functionality of both the ESCRT and autophagy machinery, highlighting a connection between Wnt, endosome, and autophagy (Romero et al., 2018). Currently, it is unclear how autophagy triggers ESCRT‐dependent GSK3 sequestration and whether this mechanism is conserved in other cell types.

Although the GSK3‐sequestration model provides a mechanistic explanation for how internalization of the signalosome stimulates Wnt/β‐catenin signaling and has been proven independently in different studies, endocytosis might activate Wnt also through additional mechanisms. In fact, several authors have observed that activation of the Wnt signal through GSK3 inhibition still requires endocytic trafficking. LiCl, a potent though not very selective GSK3 inhibitor, was unable to stabilize β‐catenin when endocytosis was concomitantly impaired in mammalian cultured cells (Blitzer & Nusse, 2006). Similarly, it was shown that activation of signaling by another GSK3 inhibitor (SB‐216763) required dynamin‐dependent endocytosis in *Drosophila* (Gagliardi et al., 2014). In yet another work, it was observed that lowering the temperature to 4˚C (which is not permissive to endocytosis) blocked β‐catenin accumulation induced by LiCl, CHIR99021, and also APC knockout (Saito‐Diaz et al., 2018). Altogether, these studies imply the possibility that endocytosis exerts at least part of its effects downstream of the destruction complex inhibition, via an as yet unidentified mechanism.

## 9 INHIBITION OF WNT THROUGH ENDOCYTOSIS

So far, we have discussed the role played by endocytosis in promoting Wnt/β‐catenin signaling. However, Wnt signaling can be also attenuated or turned off by endocytic mechanisms. Inhibition of Wnt through endocytosis entails the clearance of the receptors Lrp5/6 and Fzd from the plasma membrane, thus preventing ligand–receptor interactions. It is possible that activation and inhibition of Wnt/β‐catenin follow distinct endocytic routes. While caveolin endocytosis promotes Wnt, clathrin endocytosis may be preferentially utilized during Wnt inhibition (Figure 4). For example, the well‐characterized Wnt antagonist Dkk1 binds Lrp6 in the extracellular space, and together with the transmembrane protein Kremen promotes Lrp6 endocytosis and degradation through the endolysosomal system (Glinka et al., 1998; Mao et al., 2001, 2002). Dkk‐dependent Lrp6 internalization occurs through the clathrin pathway, as clathrin siRNA, or pharmacological inhibition of CME, prevented Dkk1 inhibition of Wnt signaling and restored nuclear β‐catenin (Yamamoto et al., 2008). Interestingly, Dkk1 also induced depalmitoylation of Lrp6, which in this form translocated to non‐DRM rafts, likely desensitizing Wnt signaling (Sada et al., 2019).

Similarly, angiopoietin‐like 4 (Angptl4), a protein involved in lipid metabolism (Li et al., 2020), was recently identified as a novel extracellular Wnt antagonist, by promoting Lrp6 internalization and lysosome‐dependent degradation (Kirsch et al., 2017). Interaction between Lrp6 and Angptl4 was not direct. Instead, syndecans bound to the N‐terminal coiled‐coil domain of Angptl4, forming a ternary complex with Lrp6 that was successively endocytosed through the clathrin pathway. It is likely that other secreted Wnt inhibitors also promote Lrp5/6 internalization through CME, like sclerostin (van Dinther et al., 2013) and the recently isolated *Xenopus* Bighead (Colozza, 2021; Ding et al., 2018). Wnt inhibition through receptor clearance may be a common mechanism, as shown by other factors, such as transmembrane E3 ubiquitin ligase Rnf43 (Koo et al., 2012) and its paralog Znrf3 (Figure 1) (Hao et al., 2012), which mediate ubiquitination of the Fzd and Lrp5/6 receptor complex, promoting its endolysosomal degradation. Intriguingly, Znrf3 activity is regulated by a cluster of four consecutive Tyr‐based internalization motifs (YXXØ), which are kept unphosphorylated by the tumor suppressor phosphatase PTPRK (protein tyrosine phosphatase receptor‐type kappa), allowing clathrin‐dependent endocytosis of ZNRF3‐Wnt receptor complexes and reducing Wnt signaling (Chang et al., 2020). Currently, it is unclear whether Rnf43 is also regulated in a similar fashion.

TMEM88, a transmembrane protein required for cardiomyocyte differentiation, is another factor recently found to inhibit canonical Wnt through endocytosis (Lee & Evans, 2019). This protein contains a PDZ‐binding motif (VWV) at the C‐terminus, required for localization to the plasma membrane and the endosomal compartment. Mutant TMEM88 lacking the PDZ binding sequence failed to localize to the plasma membrane and early endosomal antigen 1 (EEA1)‐containing endosomes, and could not inhibit Wnt signaling. TMEM88 promoted relocalization of signalosome components (including CA‐Lrp6 and β‐catenin) to MVBs, and reduced nuclear β‐catenin (Lee & Evans, 2019). Although the molecular mechanism of TMEM88 action is not entirely clear, it likely induces Wnt signalosome degradation through the endolysosomal system. Interestingly, TMEM88 inhibited Wnt signaling even in the presence of the GSK3 inhibitor CHIR99021 (Lee & Evans, 2019), reinforcing the idea that endocytic mechanisms may also modulate Wnt downstream of the destruction complex, as observed above.

## 10 WNT AS A METABOLIC REGULATOR: PROTEIN TURNOVER AND NUTRIENT SENSING

Wnt activation does not only induce a change in transcriptional activity but also directly affects several metabolic processes, including protein turnover. In fact, it has been estimated that approximately 20% of the human proteome contains GSK3 consensus motifs (S/TXXXS/T) that would promote protein degradation upon phosphorylation (Taelman et al., 2010; Xu et al., 2009). In agreement with this, inhibition of GSK3 by Wnt elicited stabilization of multiple targets, in a transcription‐independent way. This Wnt activity, which is also referred to as Wnt‐dependent stabilization of proteins (Wnt/STOP), peaks at G2/M phase and slows down protein degradation by inhibiting GSK3‐dependent polyubiquitination, increasing protein stability and cell size in preparation for cell mitosis (Acebron et al., 2014). Wnt‐dependent reduction of free available ubiquitin (through sequestration of polyubiquitinated proteins into MVBs) may play a role in the protein stabilization process (Kim et al., 2015).

The signaling axis responsible for Wnt/STOP overlaps with the canonical Wnt machinery, except that it does not require β‐catenin mediated transcription. In this alternative mode of signaling, Lrp6 phosphorylation at PPPSP sites is maximized at the G2/M phase of the cell cycle by cyclin Y and the associated CDK (cyclin‐dependent kinase)‐like L63/PFTK, which then primes Lrp6 for subsequent CK1γ activating phosphorylations (Davidson et al., 2009). BCL9, whose activity prevents clathrin‐mediated endocytosis and degradation of the signalosome, is also required for Wnt/STOP (Chen et al., 2018). The effects of Wnt/STOP on protein stability are physiologically relevant, as they regulate early vertebrate embryogenesis as well as sperm maturation (Huang et al., 2015; Koch et al., 2015), and may be leveraged in cancer treatments. In fact, in some conditions, protein catabolism represents a viable source of certain amino acids (Suraweera et al., 2012). For example, subtypes of acute leukemias or colorectal tumors can utilize proteasome‐dependent protein degradation to obtain enough asparagine. Due to this fact, these cancers become refractory to treatments with asparaginase, an enzyme that deaminates and depletes endogenous asparagine and has been long known for its therapeutic use against lymphomas (Broome, 1963a, 1963b; Rizzari et al., 2013). However, recent studies have shown that forced activation of the Wnt/STOP pathway, for example by chemical inhibition of GSK3, impairs the ability of malignant cells to utilize protein degradation to obtain asparagine and sensitize them to asparaginase treatments (Hinze et al., ,^2019^, ^2020^). Importantly, the combined treatment of asparaginase and GSK3‐specific inhibitors eliminates leukemic or colorectal cancer cells in mouse models, without affecting noncancerous cells.

In addition to regulating protein turnover, recent studies suggest that Wnt is tightly interconnected to nutrient metabolism, through the endolysosomal system. It has been noted that canonical Wnt signaling increases rapid endocytosis and endosomal trafficking, as well as uptake and lysosomal digestion of extracellular proteins such as bovine serum albumin (BSA), via protein arginine methylation (Albrecht et al., 2018). Importantly, Wnt signaling dynamically adapts to nutrient availability through S‐adenosylmethionine (SAM), a nutrient‐sensing metabolite that is generated from methionine by the one‐carbon metabolic pathway. SAM is a universal methyl donor that PRMTs use to transfer methyl groups to arginine residues (Blanc & Richard, 2017). Depletion of methionine, the SAM precursor, from the extracellular medium reduced Wnt signaling, Wnt‐induced lysosomal activity, and ingestion of extracellular proteins in cultured cells (Albrecht et al., 2019). Because PRMT1 and arginine methylation are key regulators of Wnt‐mediated sequestration of GSK3 (Albrecht et al., 2018), a reduction in methionine/SAM levels would conceivably reduce PRMT1 activity and decrease Wnt signaling. This mechanism supports a model where Wnt signaling, endosomal trafficking, and extracellular protein uptake are coordinated by nutrient levels, through the availability of methionine/SAM and arginine methylation.

## 11 WNT, NUTRIENT UPTAKE, AND MACROPINOCYTOSIS

The observed increase in extracellular BSA uptake upon Wnt3a addition to cultured cells is a result of sustained macropinocytosis (Tejeda‐Munoz et al., 2019), an evolutionarily conserved endocytic process characterized by the uptake of large amounts of extracellular fluids (pinocytosis is Greek for “cell drinking”) (Palm, 2019). Macropinocytosis is an actin‐driven process initiated by membrane protrusions forming cup‐shaped ruffles. When these ruffles fold back, fuse together, and pinch off the plasma membrane, they eventually engulf portions of the extracellular fluid with all the macromolecules contained therein, forming intracellular vesicles of varying sizes (called macropinosomes). The content of macropinosomes is either recycled or digested into lysosomes. Because of its nonselective nature, this endocytic process allows cells to ingest different types of macromolecules that serve as bulk reservoirs of nutrients such as amino acids, sugars, or lipids. As most biomass is concentrated into macromolecules, macropinocytosis represents an important route for nutrient acquisition and many cancers coopt this pathway to support their high metabolic demand even in a nutrient‐poor environment such as the tumor niche (Zhang & Commisso, 2019). Pancreatic carcinoma, for example, utilizes macropinocytosis to provide enough glutamine as well as other substances to sustain cancer cell proliferation (Commisso et al., 2013). Macropinocytosis is governed by actin cytoskeleton regulators, including the small GTPases Ras, Rab5, Rac1, and Cdc42, as well as phosphatidylinositol 3‐kinase (PI3‐kinase) and p21‐activated kinase‐1 (PAK1) (Recouvreux & Commisso, 2017). Several signaling molecules can trigger macropinocytosis by activating the Ras pathway, such as EGF and platelet‐derived growth factor (PDGF). Furthermore, oncogenic mutations in HRAS and KRAS promote higher macropinocytic activity in bladder and pancreatic cancer cells, respectively (Commisso et al., 2013; Recouvreux & Commisso, 2017). Plasma membrane V‐ATPase has recently also been shown to be an essential regulator of macropinocytosis, and its translocation to the plasma membrane is facilitated by RAS oncogenic mutations in cancer cells (Ramirez et al., 2019).

Recent discoveries have also implicated Wnt signaling in macropinocytosis. A whole‐genome shRNA screen in Ras wild‐type bladder cancer cells identified several Wnt inhibitors as suppressors of macropinocytosis (Redelman‐Sidi et al., 2018). These included DKK2, KREMEN1, NKD1, SMAD4, and MAPK9, and their depletion increased macropinocytic uptake of Bacillus Calmette‐Guerin (BCG, an attenuated strain of *Mycobacterium bovis*), concomitantly with Wnt activation. This effect was dependent on β‐catenin transcriptional activity, confirming a direct link between canonical Wnt and macropinocytosis. Stimulation of Wnt signaling with recombinant Wnt3a protein, or through knockdown of APC or Axin1 similarly increased macropinocytic activity and uptake of fluorescent dextran, a commonly used probe for macropinocytosis (Redelman‐Sidi et al., 2018). This could be inhibited by the well‐known inhibitors EIPA or IPA‐3, confirming that Wnt‐induced uptake occurred via PAK1‐dependent macropinocytosis. Furthermore, the increase in macropinocytosis was sufficient to support albumin‐dependent growth when cells were cultured in the absence of essential amino acids. In parallel, similar findings were obtained by the De Robertis group, showing that Wnt activation by different means (e.g., stimulation with Wnt3a ligand, overexpression of Dvl, etc.) triggered macropinocytosis and uptake of BSA or fluorescent dextran in cultured cells, in a way that was dependent on PRMT1 and the ESCRT component VPS4 (Tejeda‐Munoz et al., 2019). SW480 colorectal cancer cells, which have high Wnt signaling caused by the loss of APC, also displayed increased macropinocytosis.

It seems that the Wnt destruction complex actively suppresses macropinocytosis, as chemical inhibition of GSK3 (by LiCl or CHIR99021) or loss of either Axin1 or APC all promoted a strong increase in uptake of extracellular proteins by macropinocytosis and their digestion in the lysosome (Albrecht et al., 2020) (Figure 4). Axin1‐mutant hepatocellular carcinoma cells (HCC, also known as Alexander cells) displayed a strong enrichment in macropinosome‐like vesicles and increase in fluorescent dextran uptake, promptly blocked by EIPA or IPA‐3. Metabolic analysis confirmed that Wnt activation induced an increase of nutrients derived from digestion of extracellular albumin (Albrecht et al., 2020). Importantly, LiCl‐induced activation of macropinocytosis was also observed in vivo in *Xenopus laevis* embryos and was paralleled by an increase in the phosphorylated active form of PAK1 (Albrecht et al., 2020). Similarly to LiCl, stimulation with Wnt3a can also increase phosphorylated active PAK1 (Albrecht et al., 2020; M. Kim et al., 2016), which in turn phosphorylates and inhibits the neurofibromatosis type 2 (NF2) tumor suppressor Merlin (Kim et al., 2016). When Merlin is inactive, cortical patches containing actin, ezrin, and other actin remodeling proteins quickly assemble at the cell cortex following growth factor stimulation, priming cells to respond robustly to EGFR‐induced macropinocytosis (Chiasson‐MacKenzie et al., 2018). A similar mechanism may occur during Wnt signaling. Altogether, these data show that Wnt is involved in a complex metabolic rewiring that allows cells to use extracellular protein as a source of essential nutrients, and may be pivotal in sustaining the high metabolic demand of Wnt‐driven cancers.

A key observation is that macropinocytosis itself is required for Wnt signaling (Tejeda‐Munoz et al., 2019). In fact, when SW480 cells were treated with the Na^+^/H^+^ exchanger inhibitor EIPA, Wnt/β‐catenin reporter and β‐catenin nuclear accumulation were strongly inhibited (Table 1) (Tejeda‐Munoz et al., 2019). While it is still unclear how macropinocytosis feeds back positively on Wnt signaling, it possibly entails multiple mechanisms, including β‐catenin shuttling between membrane and cytoplasm. In 3T3 cells, β‐catenin was shown to localize at membrane ruffles, where macropinocytosis occurs, through interactions with the actin regulator IQGAP1 (Sharma & Henderson, 2007). β‐catenin recruitment at membrane ruffles was stimulated by GSK3 inhibition upon LiCl‐ or Wnt‐conditioned medium treatment (Johnson et al., 2009), as well as via Cdc42‐dependent phosphorylation of GSK3 Ser9 at the leading edge of migrating astrocytes (Etienne‐Manneville & Hall, 2003). From the membrane ruffling sites, β‐catenin was internalized and transported through macropinosomes that also contained APC and N‐cadherin (Sharma & Henderson, 2007). Of note, LiCl also increased trafficking of β‐catenin‐containing macropinosomes, stimulating the shuttling of β‐catenin from membrane ruffle to the nucleus via macropinocytic internalization (Johnson et al., 2009). This observation might explain why inhibition of endocytic trafficking is able to block signaling induced by GSK3 inhibition (Blitzer & Nusse, 2006; Gagliardi et al., 2014), as β‐catenin nuclear translocation would require, at least in part, membrane remodeling and internalization.

On the other hand, macropinocytosis could increase recycling and trafficking of Wnt receptors to the plasma membrane, as has already been observed for EGFR (Chiasson‐MacKenzie et al., 2018). Furthermore, Merlin was reported to act as a Wnt inhibitor both in mammalian cells and *Xenopus* embryos, by binding to Lrp6 (Kim et al., 2016; Zhu et al., 2015). Upon phosphorylation by active phospho‐PAK1, Merlin loses affinity for Lrp6, which then becomes available to transduce downstream signaling (Kim et al., 2016). Altogether, these data demonstrate the presence of an intricate regulatory feedback that connects Wnt to macropinocytosis.

## 12 CONCLUDING REMARKS

There is mounting evidence showing that Wnt does not only operate through transcriptional changes, but profoundly affects membranous compartments, protein stability, cell metabolism, and nutrient acquisition, via regulation of various endocytic mechanisms. The growing list of signaling pathways able to directly regulate nutrient uptake, to which Wnt has been added as a new member, is a clear demonstration that dysregulation of growth factors not only changes gene expression dynamics in cancer cells, but also provides a growth advantage in nutrient‐poor niches. Understanding how the connection between Wnt, endocytosis, and metabolism works at a molecular level will be of importance for developing new therapeutic approaches for treating Wnt‐driven cancers.
